# Shape-controllable synthesis of hydrophilic NaLuF_4_:Yb,Er nanocrystals by a surfactant-assistant two-phase system

**DOI:** 10.1186/1556-276X-8-518

**Published:** 2013-12-06

**Authors:** Na Zhou, Peiy Qiu, Kan Wang, Hualin Fu, Guo Gao, Rong He, Daxiang Cui

**Affiliations:** 1 Institute of Nano Biomedicine and Engineering, Key Laboratory for Thin Film and Microfabrication Technology of the Ministry of Education, Research Institute of Micro/Nano Science and Technology, Shanghai Jiao Tong University, 800 Dongchuan Road, Shanghai, 200240, People’s Republic of China

**Keywords:** Upconversion, Rare earths, Surfactant, Capping agent, Luminescence, Cytotoxicity

## Abstract

Water-soluble upconversion nanoparticles (UCNPs) were prepared by a one-pot procedure in a two-phase reacting system. Four kinds of surfactants were tested in the synthesis process as capping agent to tune size and morphology of nanocrystals. Nanoparticles (approximately 70 nm) and rods (400 nm and 2.5 μm) were synthesized, respectively. Then, Fourier transform infrared spectroscopy analysis confirmed the successful linking between UCNP surface and surfactant. Ionic liquids (ILs) and surfactants participated in synthesis process together, competing with each other to cap on UCNPs. ILs still led the competition of capping, while surfactants worked as cooperative assistants to develop functional surface. Further characterizations such as high-resolution transmission electron microscopy and X-ray diffraction indicated the changes in crystallization and phase transformation under the influence of surfactants. In addition, the growth mechanism of nanocrystals and upconversion fluorescence luminance was also investigated in detail. At last, the cytotoxicity of UCNPs was evaluated, which highly suggest that these surface-functionalized UCNPs are promising candidates for biomedical engineering.

## Background

In the past decades, lanthanide (Ln)-doped upconversion nanoparticles (UCNPs) have attracted considerable attentions in the area of solar cells, detection of heavy metal in effluent and biomedical engineering including molecular imaging, targeted therapy and diagnosis all over the world due to their distinctive chemical and optical properties [1-4]. The unnatural UC behavior, converting near-infrared radiation (typically 980 nm) to high-energy emissions, has many unique advantages in biology field, including auto-fluorescence minimization, large anti-stokes shifts and penetrating depth, narrow emission peaks, and none-blinking [1,2,5]. However, conventional downconversion (DC) emission, such as quantum dots (QDs), has some intrinsic limitations including inherent toxicity and chemical instability in the bio-system despite of their tunable size-dependent emission and high quantum yields [6,7].

The choice of the host material is a key factor for achieving efficient UC luminescence. Among all of the studied UC host materials such as oxides, fluorides, and vanadates, Ln-doped fluorides (NaLnF_4_) are considered to be the most efficient host matrices for UC emission due to its low phonon energy, which decreases the non-radiative relaxation probability and results in more efficient UC emissions [8]. Especially, a lot of research has focused on the study of NaYF_4_[7-12]. Recently, reports on NaLuF_4_ have become increasingly impressive and showed its brighter potential. For example, Li’s group have resoundingly synthesized sub-20 nm [13] and sub-10 nm [14] water-stable Lu-UCNPs, which can be an ideal choice for multimodal imaging (UCL/CT/MRI/PET) agents. Notably, the sub-20 nm NaLuF_4_ co-doped Yb^3+^and Er^3+^(Tm^3+^) show about tenfold stronger UCL emission than that of corresponding hexagonal NaYF_4_-based nanocrystals with a 20-nm diameter, forecasting NaLuF_4_ an ideal host for multimodal bio-imaging probes [14,15].

Up to date, great efforts have been devoted to the synthesis of high-quality UCNPs typically through hydrothermal reaction and thermal decomposition of RE organic precursors, two most commonly used synthetic methods. However, they still have their respective defects albeit successful in some respects. For instance, typical synthetic methods generally need complicated post surface modification to couple with functional groups for hydrophily and biocompatibility [16], which is a two-step synthesis. Recently, our group has introduced a novel oleic acid-ionic liquids (OA-ILs) dual phase synthesis method, by which hydrophilic and hydrophobic Ln-doped upconversion crystals could be selectively synthetized in a one-pot approach [17-19]. In fact, the hydrophilic products obtained by dual-phase method are poorly dispersed and easy to get aggregated in solution because of the complicated surface groups coming from ILs. In a word, one-step synthesis method can simplify the reaction procedure, while products by the two-step synthesis can have better uniformity and monodispersity. As we know that some hydrophilic agents can participate in ligand exchange reaction to endow nanomaterial with hydrophilia and good monodispersity, including sodium citrate [20], polyethylene glycol (PEG) [21], EDTA [22,23], 6-aminohexanoic acid (AA) [24], etc.

Herein, we introduced a representative surfactants into OA-ILs two-phase reaction system to improve the dispersity, by using the notion of OA-ILs two-phase approach (the advantage of one-pot strategy) and ligand exchange functionalization (the advantage of better dispersity). Sodium dodecyl sulfate (SDS) and dodecyl dimethyl benzyl ammonium chloride (DDBAC) represent anionic and cationic surfactants, while PEG and sodium citrate (Cit-Na) present non-ionic surfactants with hydroxyl and carboxyl, respectively. Cit-Na is regarded as a good chelating agent in order to prevent further aggregation of particles [22]. SDS has a comparatively high HLB (up to 40) [25], which means that it is able to provide considerable anionic hydrophilic groups. DDBAC, the positively charged quaternary ammonium salt can make itself absorbed on the surface with negative charge [26]. PEG is a polymer comes from polyhydric alcohols with relatively large viscosity. Various active groups of surfactants successfully capped onto nanocrystals surface during the synthetic process, resulting in Cit-UCNPs, SDS-UCNPs, DDBAC-UCNPs, and PEG-UCNPs. The size, morphology, phase, and emission intensity of the above four UCNPs were also investigated compared to those without surfactants (IL-UCNPs).

## Methods

### Material preparation

All RE oxides, including Lu_2_O_3_ (99.99%), Yb_2_O_3_ (99.99%), and Er_2_O_3_ (99.99%), were obtained from Aladdin Chemistry, Shanghai, China. Sodium oleate, OA, ethanol, Cit-Na, PEG, DDBAC, and SDS were purchased from Sinopharm Chemical Reagent, Shanghai, China. BmimPF_6_ was purchased from Shanghai Cheng Jie Chemical, Shanghai, China. MGC-803cells and GES-1 cells were available from the cell store of the Chinese Academy of Science, Shanghai, China. Cell culture products and reagents, unless mentioned otherwise, were purchased from GIBCO, Langley, OK, USA. Deionized water (Millipore Milli-Q grade, Billerica, MA, USA) with a resistivity of 18.2 MW cm was used throughout the synthetic and post-synthetic treatment procedures.

### Synthesis of NaLuF_4_:Yb, Er with different surfactants

RE-(oleate)_3_ complexes (RE = Lu, Yb, Er) were synthesized according to previously reported methods [15,27]. Typically, 0.78 mmol Lu(oleate)_3_), 0.2 mmol Yb(oleate)_3_, 0.02 mmol Er(oleate)_3_, and 1 mmol sodium oleate were dissolved in a small amount of OA at elevated temperature under vigorous magnetic stirring to form a homogeneous solution. Then, the solution was transferred into a 50-mL Teflon-lined autoclave, which contained 15 ml BmimPF_6_ to form a two-phase reaction system. Finally, 10 mL ethanol solutions including 0.1 mmol surfactants (Cit-Na, PEG, DDBAC, SDS) were added and the two-phase system was heated to 250°C and maintained for 24 h. The whole system was allowed to cool to room temperature. All precipitates were found in the IL phase. The particles were isolated by means of centrifugation at a speed of 8,500 rpm. The products were washed with ethanol under ultrasonic conditions for several times to remove the residue. Finally, the products were dried at 70°C under vacuum overnight.

### Characterization

The morphology of the nanocrystals was determined by scanning electron microscopy (FEI-Sirion 200, Hillsboro, OR, USA) and transmission electron microscopy (JEM 2100 F, JEOL Ltd., Akishima-shi, Japan). Powder X-ray diffraction (XRD) measurements were conducted on a X-ray diffractometer (Rigaku, Shibuya-ku, Japan) with Cu Kα radiation at 1.540 Å at a scanning rate of 4° min^-1^ in the 2*θ* range from 10° to 70°. Fourier transform infrared spectroscopy (FTIR) analysis was carried out on an EQUINOX 55 spectrometer (Bruker, Karlsruhe, Germany). UC fluorescence spectra were characterized using a Fluorolog-3 spectrofluorometer (JobinYvon, Palaiseau, France) at room temperature. Thermogravimetric analysis (TGA) analyses were carried out on a Pyris 1 TGA instrument (PerkinElmer, Waltham, MA, USA).

### Cell culture and cytotoxicity assay

Human gastric cancer MGC-803 cells and human normal gastric GES-1 cells were grown in RPMI-1640 medium with 10% fetal bovine serum (FBS) and 1% penicillin at 37°C in a humidified 5% CO_2_atmosphere. MGC-803 cells and GES-1 cells (4 × 10^3^ cells/well) were seeded in 96-well plates and incubated overnight. After being rinsed with PBS, the cells were incubated with varying concentrations of Cit-Na modified NaLuF4:Yb, Er UCNPs (0, 5, 10, 20,40, 80 μg/mL) prepared above for 12 h at 37°C in the dark under the same conditions. Cell viability was determined by methyl thiazolyl tetrazolium (MTT) assays. MTT (20 μL, 5 mg/mL) was added to each well, and then, the plate was incubated for another 4 h. The medium was removed, and the formazan crystals formed were dissolved in 150 μL of dimethylsulfoxide (DMSO). The absorbance at 570 nm was measured with a standard microplate reader (Scientific Multiskan MK3, Thermo, Waltham, MA, USA). Results were calculated as percentages relative to control cells. Data are mean ± standard deviation from three independent experiments.

## Results and discussion

**Figure 1:**
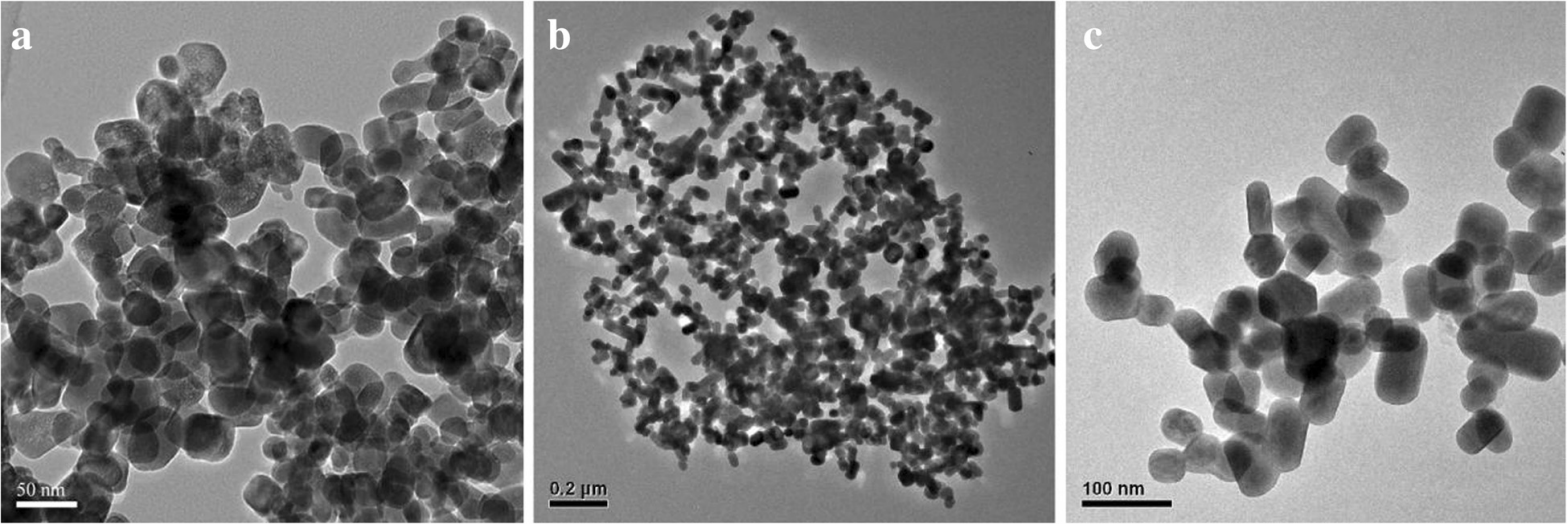
TEM image of (a) ILs-UCNPs, (b,c) Cit-UCNPs.

**Figure 2:**
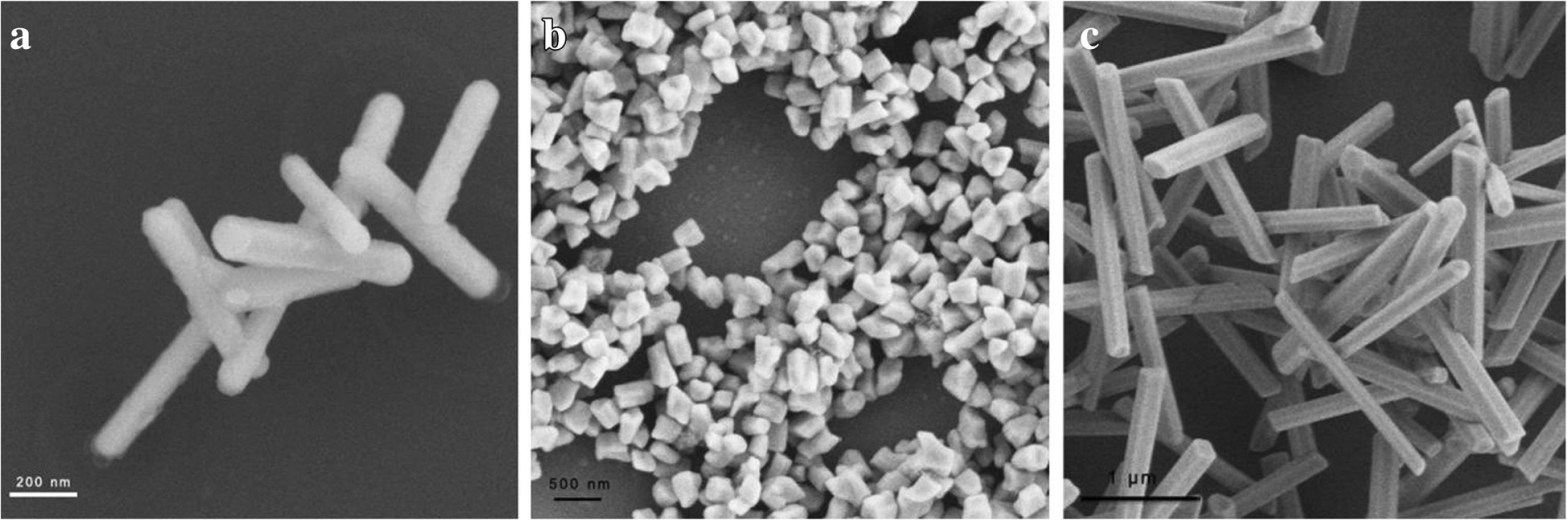
SEM images of (a) SDS-UCNPs, (b) DDBAC-UCNPs, and (c) PEG-UCNPs.

In Figure 1a, the IL-capped products (IL-UCNPs) were poorly dispersed on the substrate with diverse shapes and a wide range of size distribution. Due to its surface capped with long chains from ILs, the ILs-UCNPs were hydrophilic but not easily dispersed in polar solvents even water or ethanol19]. Figure 1b,c showed the citrate capped UCNPs (Cit-UCNPs) with near spherical shape, which had a better dispersibility and narrower size distribution compared with ILs-UCNPs (Additional file 1: Figures S1b and S2b). Cit-UCNPs, with an average size of 71 nm, which was larger than IL-UCNPs (average size is about 30 nm). Figure 2 showed SEM images of SDS, DDBAC, and PEG capped NaLuF_4_ nanorods, respectively. The lengths of SDS-UCNPs and DDBAC-UCNPs were nearly 400 to 500 nm, and the latter were stockier than the former. Especially, PEG capped NaLuF_4_ had transformed into microscale rods with an average length up to 2.5 μm. According to high-resolution transmission electron microscopy images of an individual particle or a rod, except for IL-UCNPs, the other four UCNPs were all with a interplanar distance of about 5.0 Å (Additional file 1: Figures S2a, S3a, S4a, S5a), corresponding to the (100) lattice planes of the hexagonal-phase NaLuF_4_, indicating that the preferred growth direction of the hexagonal phase NaLuF_4_ nanorods is along the (100) orientation. While Additional file 1: Figure S1a showed an interplanar distance of nearly 3.1 Å, attributed to the (111) lattice plane of cubic phase. This can be understood from the growth mechanism. As is known to all that the formation of a particle includes initial production, subsequent growth, and final stabilization of nuclei [4]. Particle size is mainly determined by nucleation rate and a higher nucleation rate leads to a smaller particle size. From this viewpoint, we think that the nucleation rates differ when using different surfactant. Nucleation of a crystal includes the diffusion of ions onto the surface of a growing crystal and their subsequent incorporation in the structure of the crystal lattice. The F^-^ anion must compete with the surfactant chelators to transform Ln-complex into NaLuF_4_ lattice. A small chelate constant (lg β) would benefit the combination of F^-^ and Ln^3+^ ions resulting in the NaLuF_4_ lattice [28]. According to coordination chemistry, the chelate constants increase for sodium citrate, SDS, DDBAC, and PEG according to priority [27], resulting in gradually increasing size of UCNPs. Another reason may be attributed to the diverse viscosity of interface of dual phase system after adding surfactant [29].

To evaluate the ligand stability in each sample, TGA was performed (Additional file 1: Figures S1c, S2c, S3c, S4c, S5c). TGA curves showed two weight loss stages in the range of 20°C to 900°C. The first weight loss stage in the temperature range of 20°C to 200°C was due to the loss of absorbed water. The second stage from 200°C to 900°C was attributed to the combustion of the organic groups in the samples. A common feature was that weight of each sample decreased rapidly at 600°C to 700°C. Additionally, when temperature reached 600°C, the weight loss was still less than 10% of the total weight, indicating good stability of each ligand linking. Notably, Cit-Na had shown priority in chelate ability, whose weight loss was only 1.82% until temperature risen up to 900°C. Based on EDX spectrums (Additional file 1: Figures S1d, S2d, S3d, S4d, S5d), fluorine had occupied majority weight of UCNPs, demonstrating that the lead role of capping agent was still ILs, and other surfactants worked as cooperative assistants to develop functional surface.

**Figure 3:**
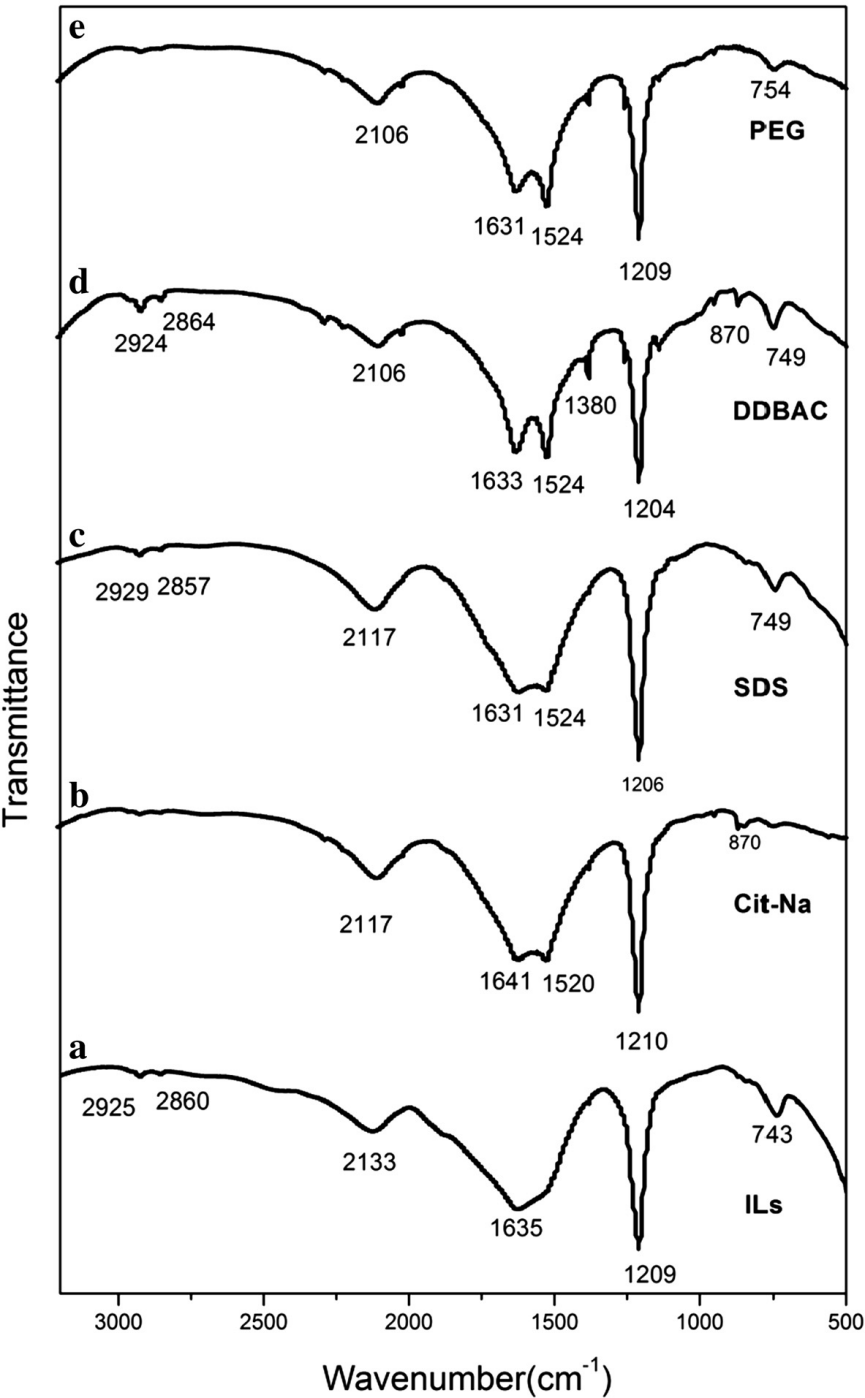
FTIR spectra of UCNP samples: (a) IL-UCNPs, (b) Cit-UCNPs, (c) SDS-UCNPs, (d) DDBAC-UCNPs, and (e) PEG-UCNPs.

The successful ligand links between surfactants and surface of UCNPs were further verified by FTIR spectroscopy. Figure 3 showed the FTIR spectra of the five UCNP samples. The transmission band peaks at approximately 2,930 and 2,854 cm^-1^ can be assigned to the asymmetric and symmetric stretching vibrations, respectively. However, these features were lost in the spectrum of the Cit-UCNPs sample, suggesting the disappearance of the –CH_2_-CH_2_– groups. What is more, bands peaks at 1,641 and 1,520 cm^-1^ belonged to the C = O vibrations, indicating the presence of carboxylic groups in Cit-UCNPs. Band peak at 1,206 cm^-1^ in Figure 3 (c) suggested that the sulfonic acid groups have been attached in the surface. In Figure 3 (d), band peaks at 2,924, 1,532, and 749 cm^-1^ indicate the presence of phenyl group. Peak at 1,524 cm^-1^ in Figure 3 (e) could indicate new groups had been attached. On the basis of the above described FTIR results, it can be deduced that the active groups of surfactants capped successfully onto UCNP surface during the synthetic process though part of surface still linked with long alkyl chains from ILs. As a consequence, ILs and surfactants participate synthesis process together as capping agents, competing with each other to cap for UCNPs.

**Figure 4:**
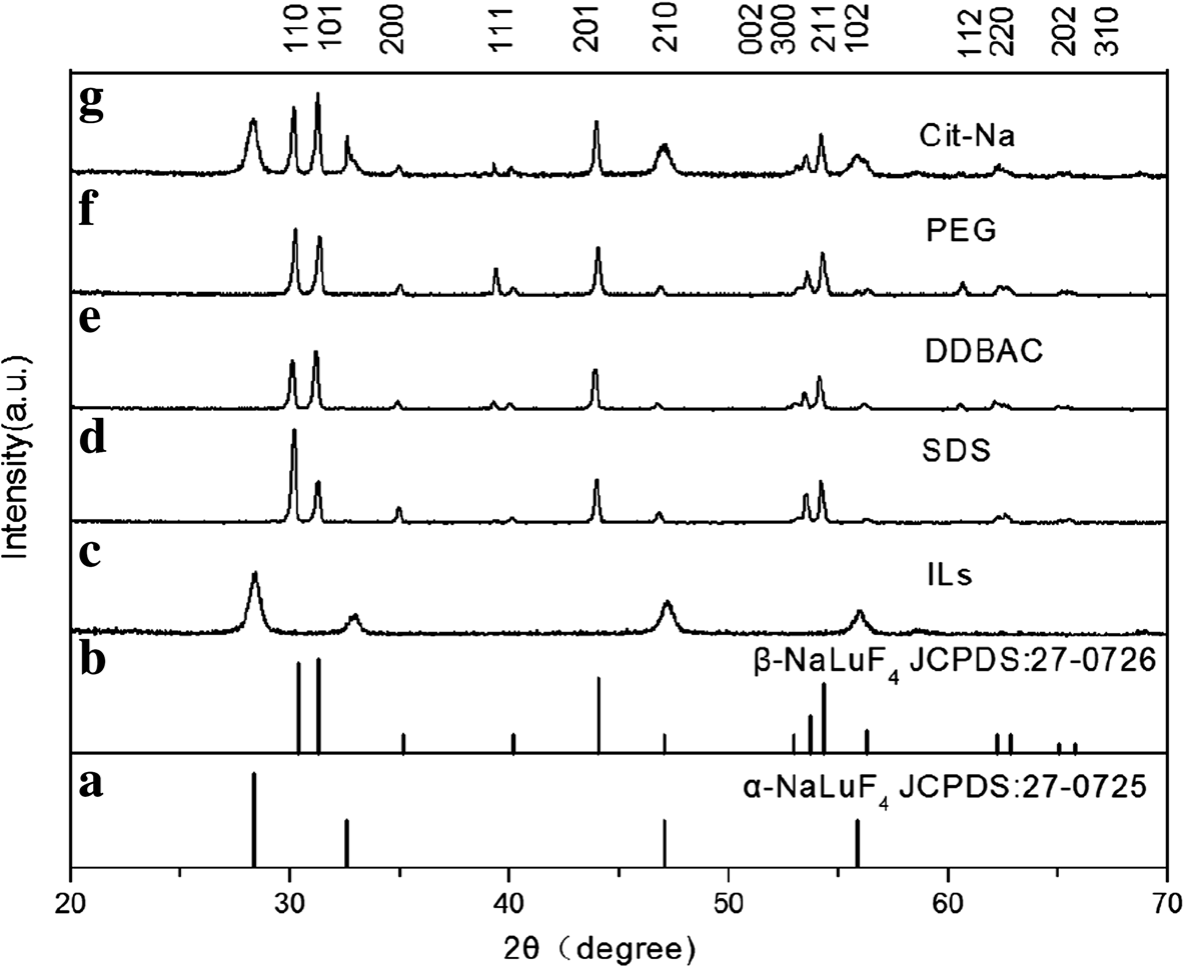
**XRD patterns of the NaLuF_4_ samples. (a)** Standard data of cubic phase (JCPDS:27–0725), **(b)** standard data of hexagonal phase (JCPDS:27–0726), **(c)** IL-UCNPs, **(d)** SDS-UCNPs, **(e)** DDBAC-UCNPs, **(f)** PEG-UCNPs, and **(g)** Cit-Na-UCNPs.

The crystallization of the ILs-UCNPs was investigated by XRD analysis (Figure 4). The peak positions and intensities correlate well with those calculated for the cubic phase NaLuF_4_ (JCPDS: 27–0725), whose morphology and size also agreed with cubic particles. The XRD patterns for the SDS, DDBAC, and PEG capped NaLuF_4_ can be indexed as single-phase hexagonal NaLuF_4_ (JCPDS: 27–0716), while the cubic and hexagonal phase co-exist as exemplified in Figure 4 (g) for those prepared with citrate. What is more, the SAED patterns of SSD, DDBAC, and PEG capped UCNPs (Additional file 1: Figures S3b, S4b, and S5b) can be readily indexed as the hexagonal phase NaLuF_4_ with single-crystalline nature, which was also well consistent with the XRD analysis. It is well known that hexagonal UCNPs generally have larger size than cubic phase, which is also corresponded to the XRD results. Therefore, the role of surfactant was not simply limited to surface ligand regulation or as a morphology controlling agent. The XRD analysis on the crystal-phase controlling capacity of different surfactants showed that the addition of SDS, DDBAC, and PEG were more effective for the crystal-phase transformation from cubic to hexagonal. This might be relevant to the co-organization of dual phases or a highly cooperative self-assembly process between organic and inorganic components [29-31].

**Figure 5:**
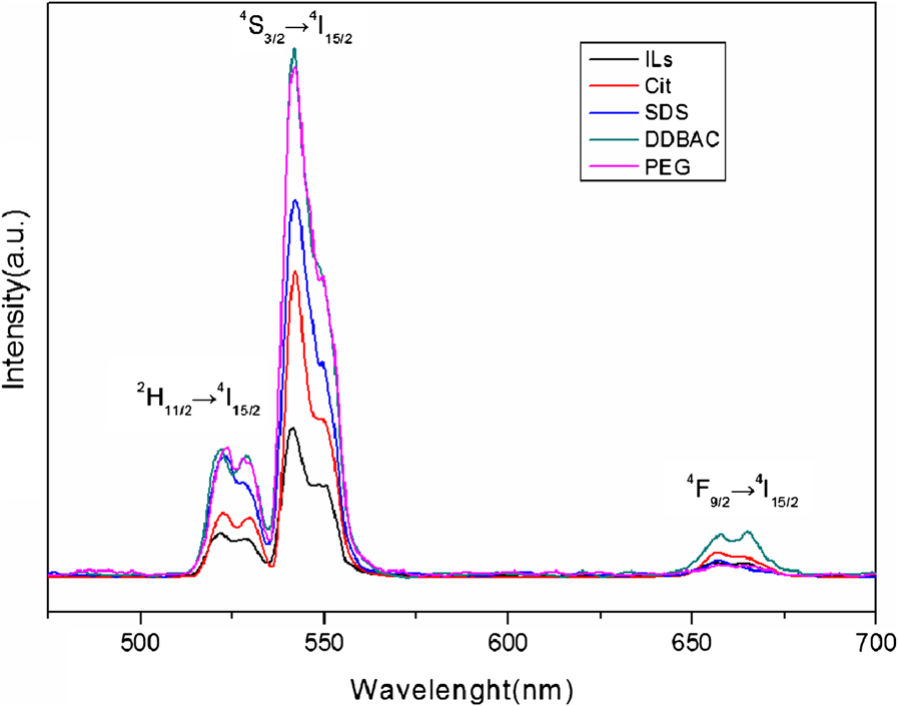
Room-temperature upconversion luminescence spectra of NaLuF_4_ powder.

**Figure 6:**
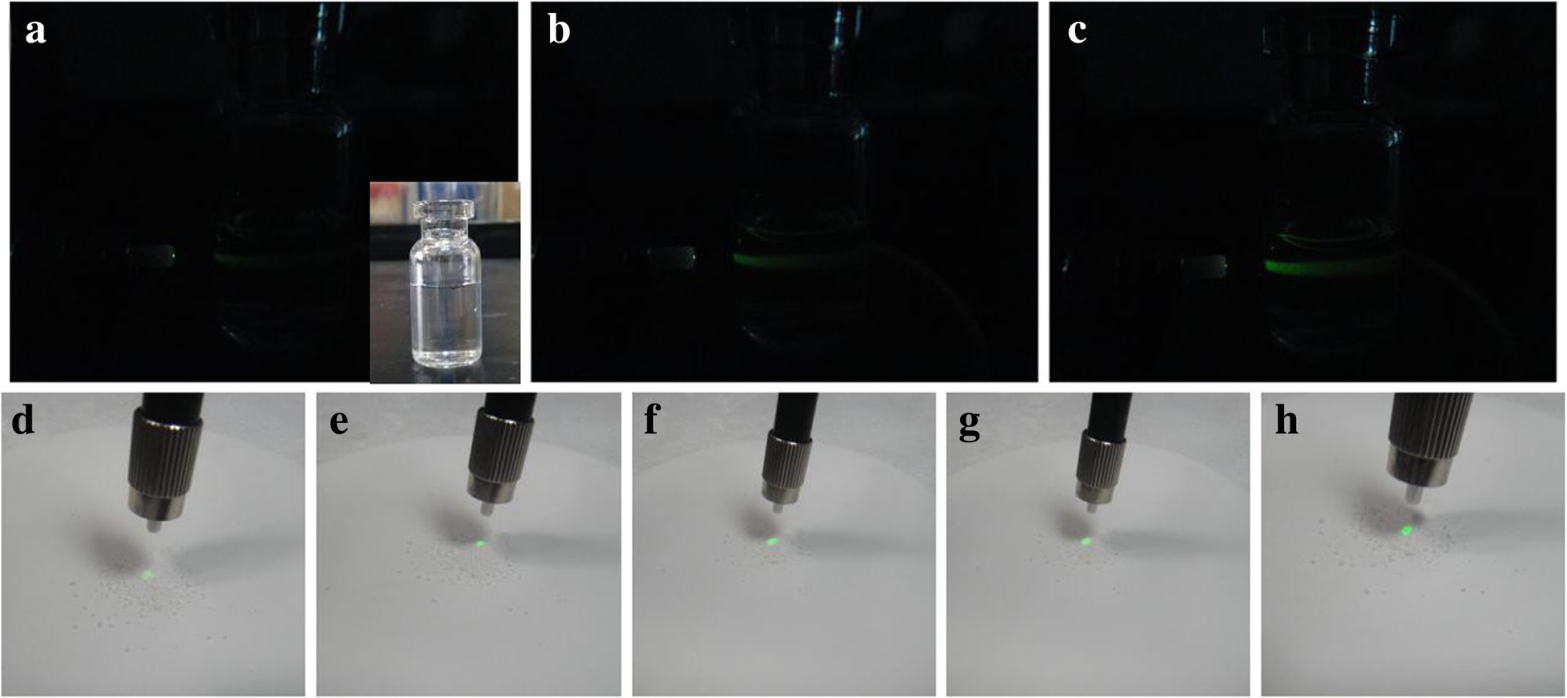
**The photograph of green UCL emissions. (a,b,c)** the photograph of 80 μg/mL colloidal solution of as-prepared ILs-UCNP, Cit-UCNP, and SDS-UCNP samples dispersed in ethanol in dark field, the insert in **(a)** displays solution in bright field **(d,e,f,g,h)** the photograph of the five kinds of UCNPs powder in bright field (Under the excitation of 980-nm laser diode with power density of 4 W/cm^2^).

Furthermore, the upconversion luminescent (UCL) properties of ILs-UCNPs, Cit-UCNPs, SDS-UCNPs, DDBAC-UCNPs, and PEG-UCNPs were investigated. Figure 5 showed the UCL spectrum of the five kinds of UCNPs powder under excitation at 980 nm (power ≈ 4 W/cm^2^). UCL peaks were all at 525, 540, and 655 nm, which can be assigned to the ^2^H_11/2_ → 4I_15/2_, ^4^S_3/2_ → ^4^I_15/2_, and ^4^ F_9/2_ → ^4^I_15/2_ transitions of erbium, respectively. The peak positions of these products were nearly the same, but the peak intensities were quite different. It is obvious that the fluorescence intensity for DDBAC-NaLuF_4_ and PEG-NaLuF_4_ was the strongest among five while ILs-NaLuF_4_ is the weakest. It is probably because the β-NaREF_4_ UCNPs provide over an order of magnitude stronger fluorescence than its corresponding cubic form [6]. On the other hand, owing to the larger surface quenching sites, smaller nanocrystals may suppress UC luminescence by enhanced nonradiative energy transfer processes of the luminescent lanthanide ions [4]. Compared to those tiny particles, the rod-like products have a relatively larger size and smaller ratio surface, leading to less surface defects. The green emissions were observed in both solution and powder (Figure 6), indicating the microrods could be applied potentially in imaging field or luminescent materials.

**Figure 7:**
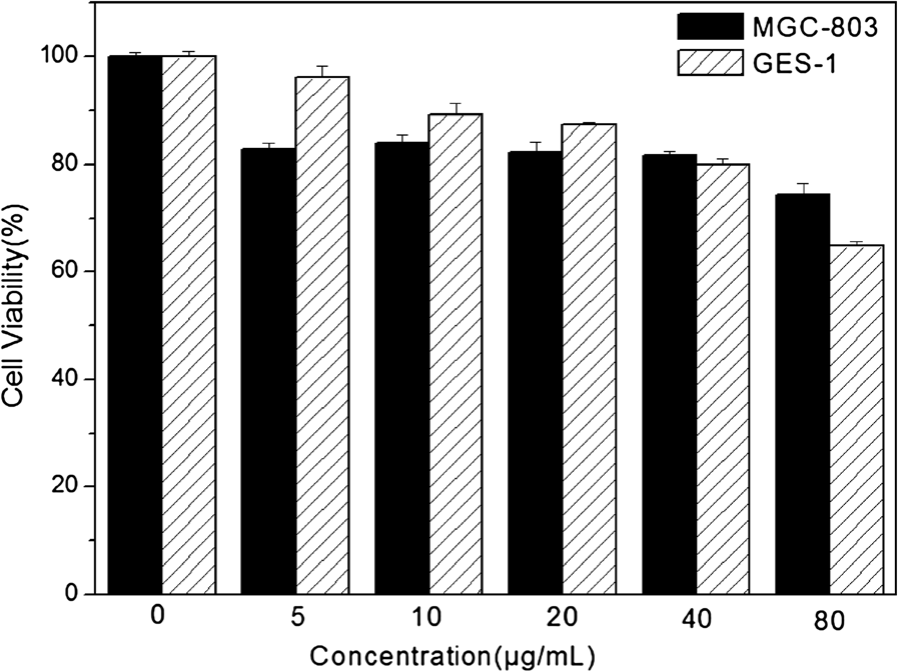
**Cytotoxicity of Cit-NaLuF_4_.** Cell toxicity was determined by MTT assay using MGC-803 cells and GES-1 cells incubated with 0 to 80 μg/mL Cit-NaLuF4 for 24 h at 37°C in the dark. Data represents mean ± SD (*n* = 5).

To evaluate the cytotoxicity of Cit-NaLuF_4_:Yb,Er nanocrystals [32], MTT assays were performed on MGC-803 cells and GES-1 cells incubated with 0 to 80 μg/mL Cit-NaLuF4 for 24 h at 37°C (Figure 7). The viability of untreated cells was assumed to be 100%. No significant difference in cell viability was observed when the concentrations of Cit-NaLuF_4_ ranged from 5 to 40 μg/mL. Even though the concentration goes up to 80 μg/mL, cell viabilities were still over 75%. On the other hand, compared with their counterpart, GES-1 cells manifested higher cell viability with a lower concentration of Cit-NaLuF_4_:Yb,Er, while lower cell viability with a higher concentration of UCNPs. This phenomenon might come to an idea that the capability of antiadversity of normal cells is stronger than that of cancer cells when incubated with a relatively high concentration of UCNPs in certain range. In light of the low cytotoxicity, Cit-NaLuF_4_:Yb,Er could be an ideal fluorescent probe for further biological applications.

## Conclusions

In summary, water-soluble NaLuF_4_:Yb,Er nanocrystals were synthesized via a simple IL-assisted dual-phase method. Surfactants were added into reaction system as capping agents to endow UCNPs with functional groups in one-step synthesis. According to SEM and TEM images, the presence of surfactants could regulate size and morphology of nanocrystals from 20- to 30-nm nanoparticles to microrods with diverse sizes. What is more, the dispersity of UCNPs was improved, accompanied with narrower particle size distribution. The FTIR analysis confirmed that the active groups had been successfully attached into the surface of UCNPs even though they had to compete with ILs. Then XRD analysis revealed that Cit-UCNPs were co-existing α and β phase, while SDS, DDBAC, and PEG functional nanocrystals have transformed into microrods with pure β phase, indicating the achievement of simultaneous phase and shape control in one step. Moreover, under the excitation of a 980-nm laser diode, visible green light emissions were observed in both solution and powder. Based on the UCL spectra, the emission intensity increased dramatically after adding surfactants. Finally, cytotoxicity of Cit-NaLuF_4_:Yb,Er was evaluated, showing prepared UC nanocrystals own good biocompatibility, indicating its potential applications in biomedical engineering in near future. Further work will focus on application of prepared NaLuF_4_:Yb,Er nanoparticles in bio-imaging, such as fluorescent imaging of cancer cells and targeted therapy *in vivo*.

## Competing interests

The authors declare that they have no competing interests.

## Authors’ contributions

NZ designed the experiment, analyzed results, and drafted the manuscript. PQ offered technical supports. NZ, PQ, KW, HF, GG, RH, and DC participated in revising the manuscript. All authors read and approved the final manuscript.

## Supplementary Material

Additional file 1: Figure S1 **(a)** High-resolution TEM image, **(b)** size distribution **(c)** TGA, **(d)** EDX spectrum of ILs-NaLuF_4_:Yb,Er. **Figure S2. (a)** High-resolution TEM image, **(b)** size distribution **(c)** TGA, **(d)** EDX spectrum of Cit-NaLuF_4_:Yb,Er. **Figure S3. (a)** High-resolution TEM image, **(b)** SAED pattern **(c)** TGA, **(d)** EDX spectrum of SDS-NaLuF_4_:Yb,Er. The inset of **(a)** shows the corresponding TEM image. **Figure S4. (a)** High-resolution TEM image, **(b)** SAED **(c)** TGA, **(d)** EDX spectrum of DDBAC-NaLuF_4_:Yb,Er. The inset of **(a)** shows the corresponding TEM image. **Figure S5. (a)** High-resolution TEM image, **(b)** SAED **(c)** TGA, **(d)** EDX spectrum of PEG-NaLuF_4_:Yb,Er. The inset of **(a)** shows the corresponding TEM image. Click here for file
