# Supplementary material for: Shape-controllable synthesis of hydrophilic NaLuF4:Yb,Er nanocrystals by a surfactant-assistant two-phase system
Source: Nanoscale Res Lett. 2013 Dec 6;8(1):518. doi: 10.1186/1556-276X-8-518 (PMC4029583; doi:10.1186/1556-276X-8-518)
Supplement: Additional file 1: Figure S1 — (a) High-resolution TEM image, (b) size distribution (c) TGA, (d) EDX spectrum of ILs-NaLuF4:Yb,Er. Figure S2. (a) High-resolution TEM image, (b) size distribution (c) TGA, (d) EDX spectrum of Cit-NaLuF4:Yb,Er. Figure S3. (a) High-resolution TEM image, (b) SAED pattern (c) TGA, (d) EDX spectrum of SDS-NaLuF4:Yb,Er. The inset of (a) shows the corresponding TEM image. Figure S4. (a) High-resolution TEM image, (b) SAED (c) TGA, (d) EDX spectrum of DDBAC-NaLuF4:Yb,Er. The inset of (a) shows the corresponding TEM image. Figure S5. (a) High-resolution TEM image, (b) SAED (c) TGA, (d) EDX spectrum of PEG-NaLuF4:Yb,Er. The inset of (a) shows the corresponding TEM image. [file 1556-276X-8-518-S1.doc]

**Supporting data**

**Shape Controllable Synthesis of Hydrophilic** **NaLuF_4_:Yb,Er Nanocrystals by a Surfactant-assistant Two-Phase System**

Na Zhou, Peiyu Qiu, Rong He, Kan Wang, Hualin Fu, Guo Gao and Daxiang Cui

**
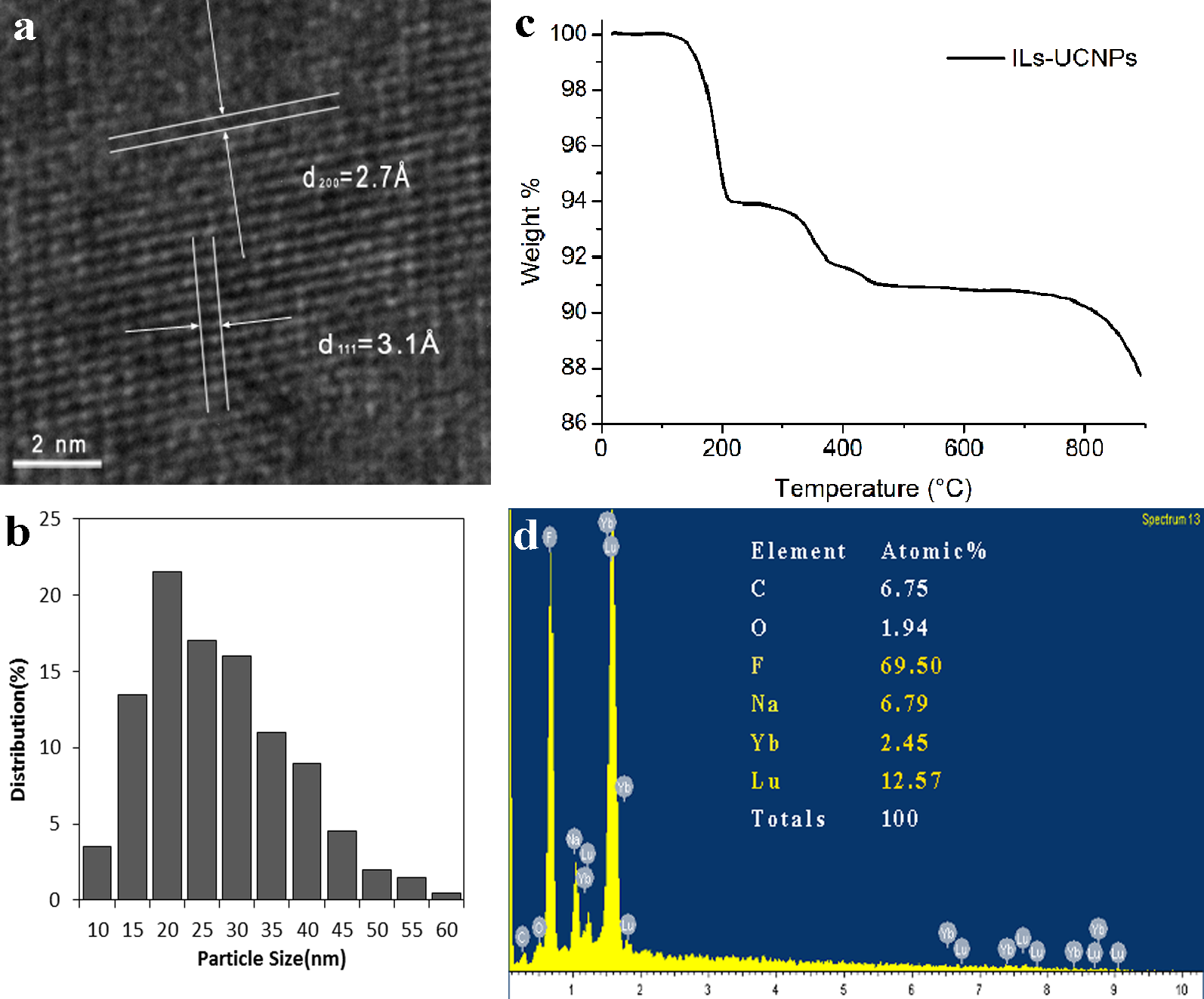
**

**Figure S1.** (a) High-resolution TEM image, (b) size distribution (c) TGA, (d) EDX spectrum of ILs-NaLuF_4_:Yb,Er


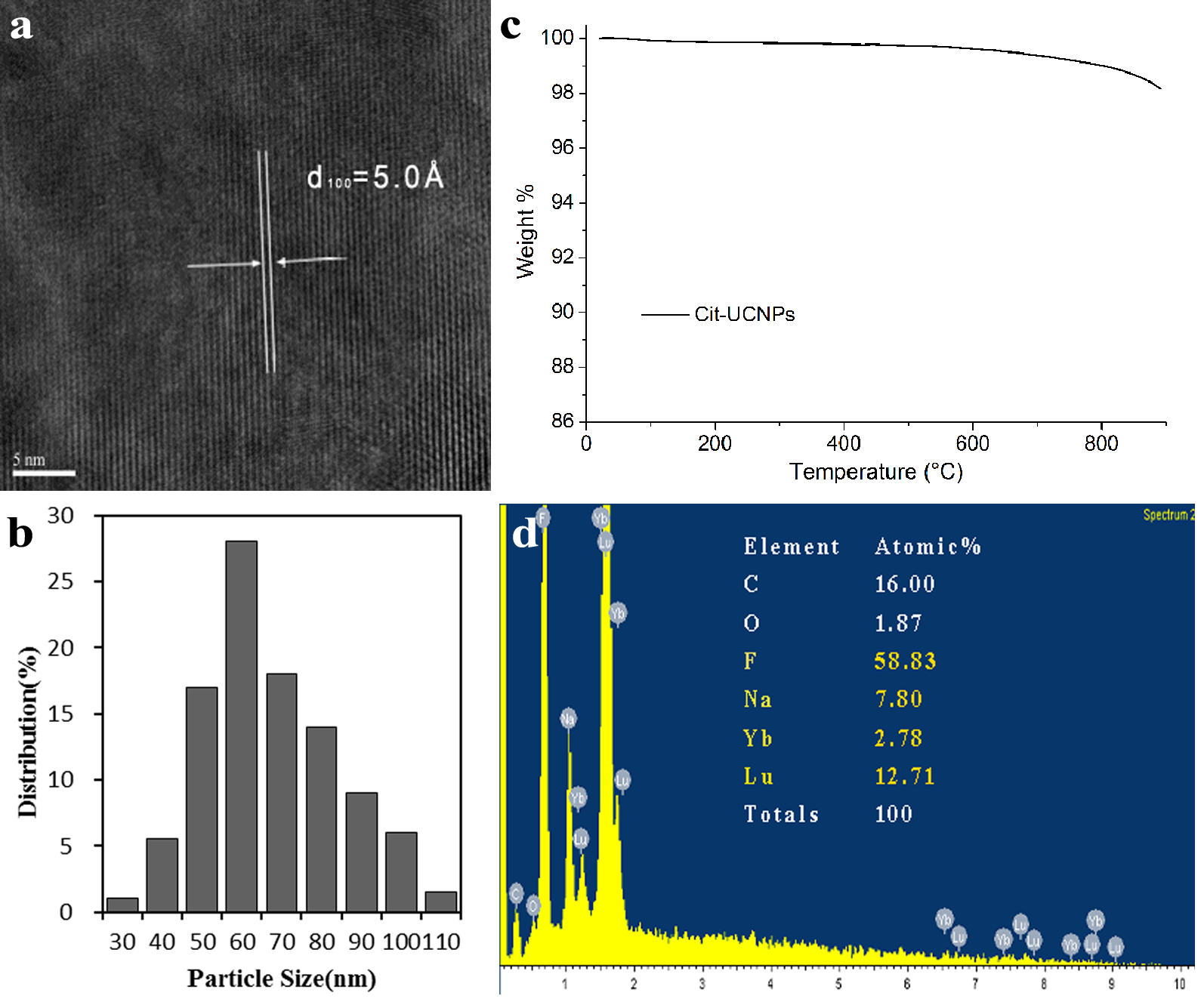


**Figure S2.** (a) High-resolution TEM image, (b) size distribution (c) TGA, (d) EDX spectrum of Cit-NaLuF_4_:Yb,Er


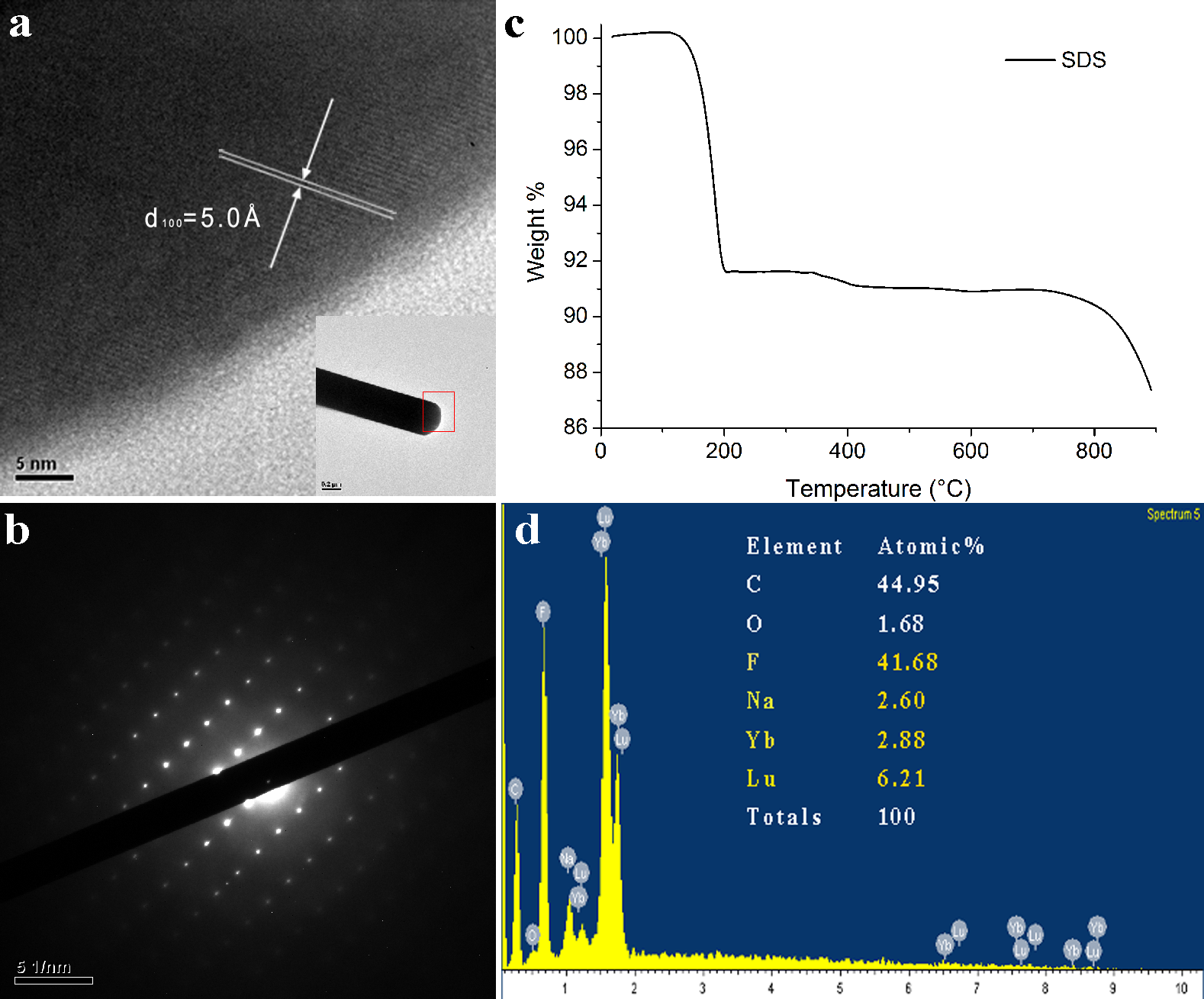


**Figure S3.** (a) High-resolution TEM image, (b) SAED pattern (c) TGA, (d) EDX spectrum of SDS-NaLuF_4_:Yb,Er. The inset of (a) shows the corresponding TEM image.


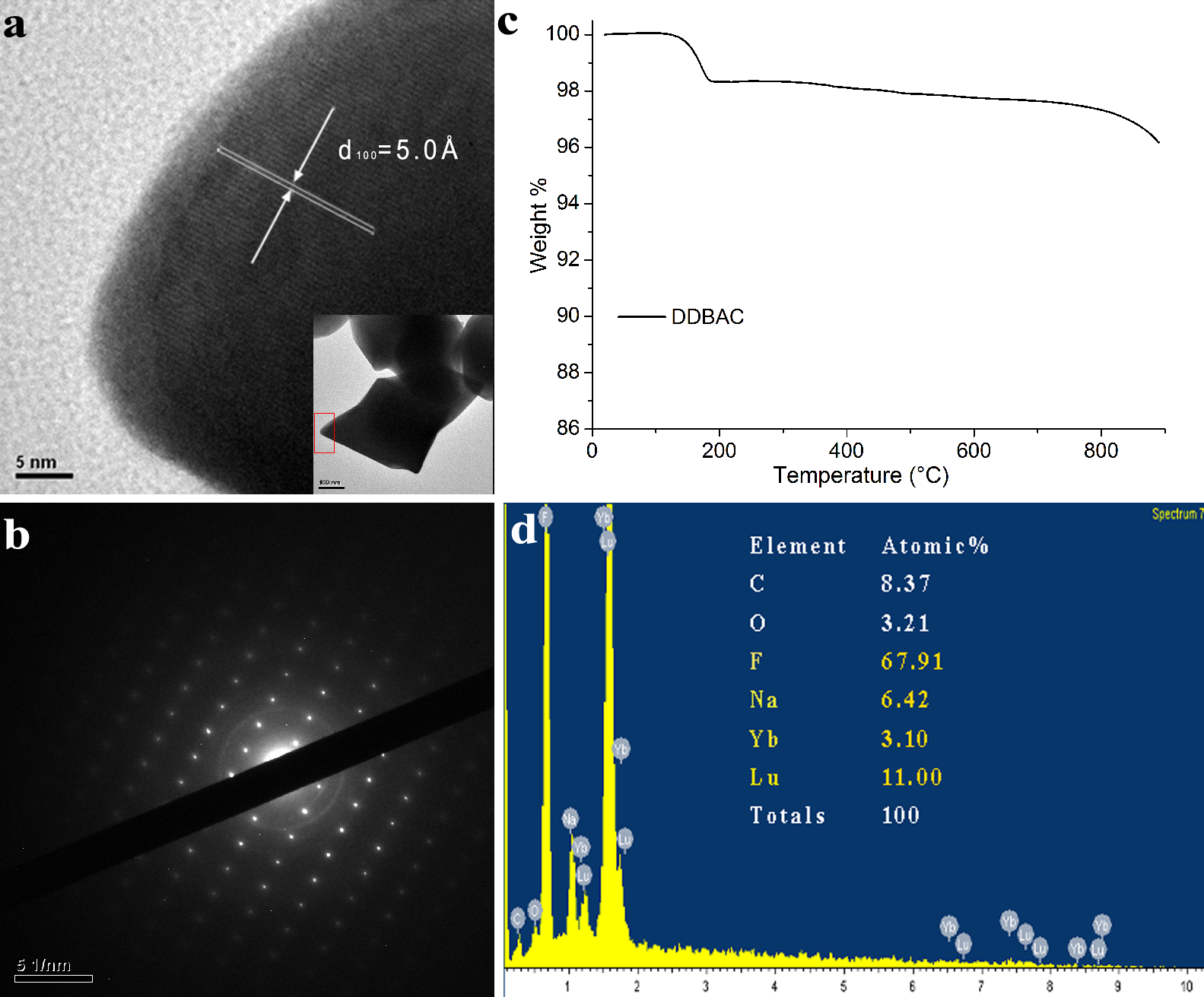


**Figure S4.** (a) High-resolution TEM image, (b) SAED (c) TGA, (d) EDX spectrum of DDBAC-NaLuF_4_:Yb,Er. The inset of (a) shows the corresponding TEM image.


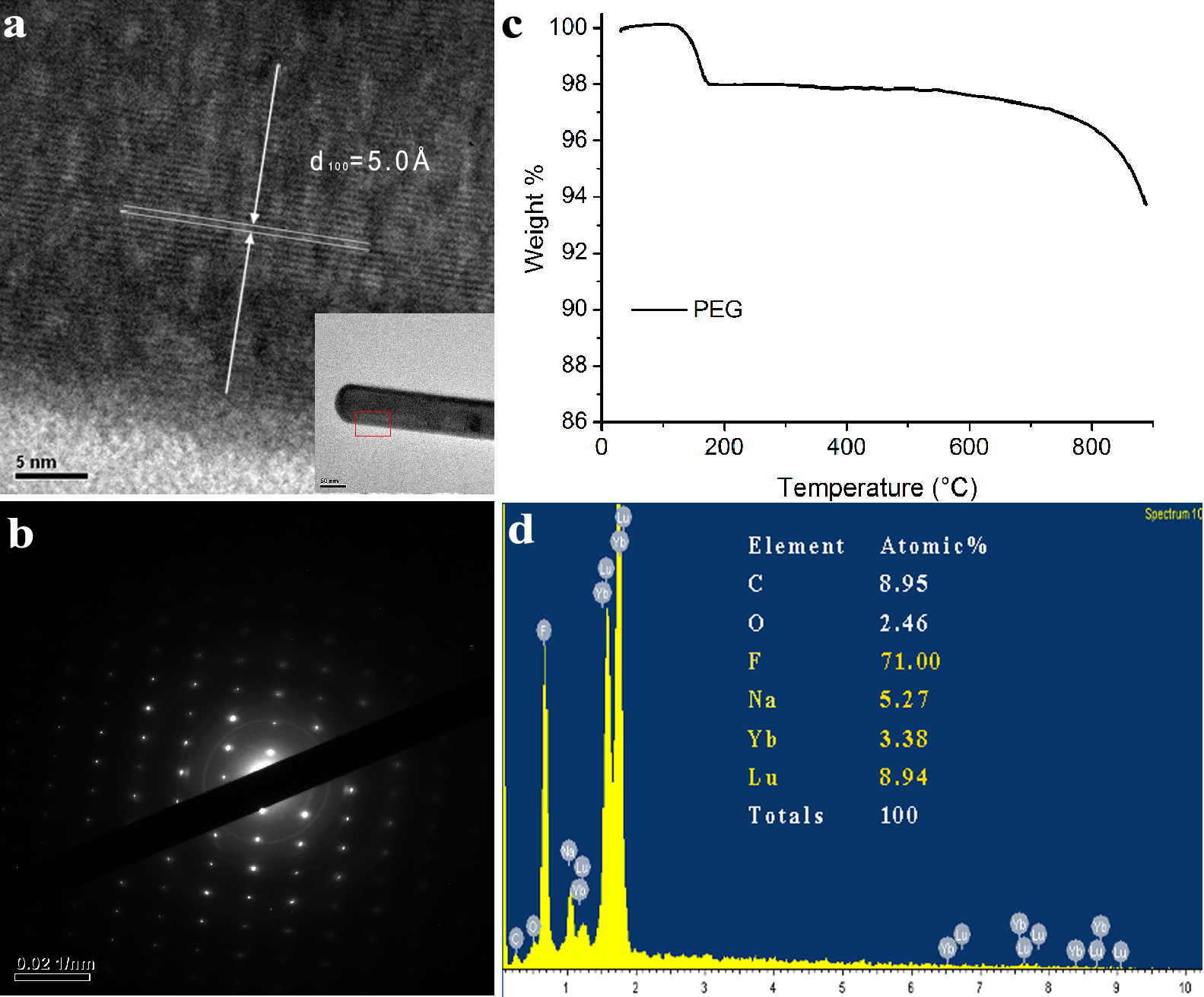


**Figure S5.** (a) High-resolution TEM image, (b) SAED (c) TGA, (d) EDX spectrum of PEG-NaLuF_4_:Yb,Er. The inset of (a) shows the corresponding TEM image.
